# Diagnostic Dilemma: Incipient Non-Arteritic Anterior Ischemic Optic Neuropathy vs. Steroid-Responsive Inflammatory Optic Neuropathy

**DOI:** 10.22336/rjo.2025.94

**Published:** 2025

**Authors:** Aahan Shah, Mary Stephen, Amit Kumar Deb, Kalyan Basa

**Affiliations:** Department of Ophthalmology, Jawaharlal Institute of Postgraduate Medical Education and Research (JIPMER), Puducherry, India

**Keywords:** non-arteritic anterior ischemic optic neuropathy, inflammatory optic neuropathy, steroid trial, crowded disc, diagnostic algorithm, BP = blood pressure, CBC = complete blood count, CT = computed tomography, FFA = fundus fluorescein angiography, HRCT = high-resolution CT, NAION = non-arteritic anterior ischemic optic neuropathy, OCT = optical coherence tomography, ONSD = optic nerve sheath diameter, OD = right eye, OS = left eye, RAPD = relative afferent pupillary defect, RNFL = retinal nerve fiber layer, VDRL = Venereal Disease Research Laboratory

## Abstract

**Objective:**

To highlight the diagnostic challenge of differentiating incipient non-arteritic anterior ischemic optic neuropathy (NAION) from inflammatory optic neuropathy in resource-limited settings.

**Methods:**

A male in his mid-50s with four days of painless progressive left-eye vision loss. Examination: OD crowded disc (CDR 0.3), OS 360°-disc edema (OCT RNFL 182 μm), Grade 1 RAPD, 360° visual field constriction. Investigations: OCT 30-2 fields, FFA (delayed disc filling, late leakage), HRCT brain/orbit, B-scan (ONSD 6.02 mm), labs (BP 126/82, PP2BS 146 mg/dL, elevated lipids and ESR, nonreactive VDRL). Treatment: aspirin 75 mg OD + prednisolone 1 mg/kg OD for a one-week trial.

**Results:**

At one week: OS edema resolved (RNFL 104 μm), vision improved to 6/9 OS, RAPD resolved; steroids tapered over 10 days. At two weeks: OS 6/6, fields markedly improved. At 10 weeks: maintained on pred 5 mg OD + aspirin; no recurrence.

**Discussion:**

The case highlights the complexity of distinguishing incipient NAION from steroid-responsive inflammatory optic neuropathy, particularly when clinical and angiographic features overlap. The dramatic improvement following a short steroid trial suggested an underlying inflammatory component despite the presence of structural risk factors for NAION. This therapeutic response emphasizes the diagnostic value of monitored steroid trials in ambiguous presentations. In resource-limited environments, such an approach may offer a pragmatic adjunct to standard diagnostic protocols.

**Conclusions:**

Rapid steroid response favored an inflammatory component over pure NAION. A crowded OD disc predisposed to NAION underscores the necessity for nuanced, individualized management. Therapeutic trials may aid diagnosis where advanced tests are inaccessible.

## Introduction

Non-arteritic anterior ischemic optic neuropathy (NAION) remains the most common cause of acute optic nerve-related visual loss in adults over 50. Yet, its early stages may be clinically indistinguishable from inflammatory optic neuropathies. In settings lacking advanced neuroimaging or electrophysiology, this overlap poses significant diagnostic challenges [**1-3**]. The crowded optic disc, a well-recognized anatomical risk factor for NAION, can further confuse the presentation when accompanied by disc edema and field constriction. Conversely, inflammatory optic neuropathies often exhibit steroid responsiveness, but may present without pain or systemic symptoms. Fundus fluorescein angiography, while helpful, may still yield inconclusive patterns in early disease. These diagnostic uncertainties make timely management decisions difficult and potentially vision-limiting [**4,5**]. This report describes a case in which a structured therapeutic steroid trial was a key determinant in clarifying the underlying pathology. The case underscores the need for adaptable, evidence-supported diagnostic strategies in ophthalmic practice.

## Case Presentation

Patient Demographics and Chief Complaint

A male in his 50s presented with painless, progressive diminution of left-eye vision for four days. No ocular pain, flashes, floaters, or metamorphopsia.

Past Medical, Ocular, and Systemic History

No diabetes, hypertension, CAD, CKD, or cerebrovascular disease.

No neurological symptoms, prior ocular surgeries, or systemic inflammatory conditions.

Ocular Examination

Visual Acuity: OD 6/12 → 6/9 (–3.50 D); OS 6/24 → 6/12 (–4.50 D)

Pupils: Grade 1 RAPD OS

Anterior Segment: Clear cornea, deep quiet AC, no cells/flare, clear lens, no NVI.

**Fig. 1:**
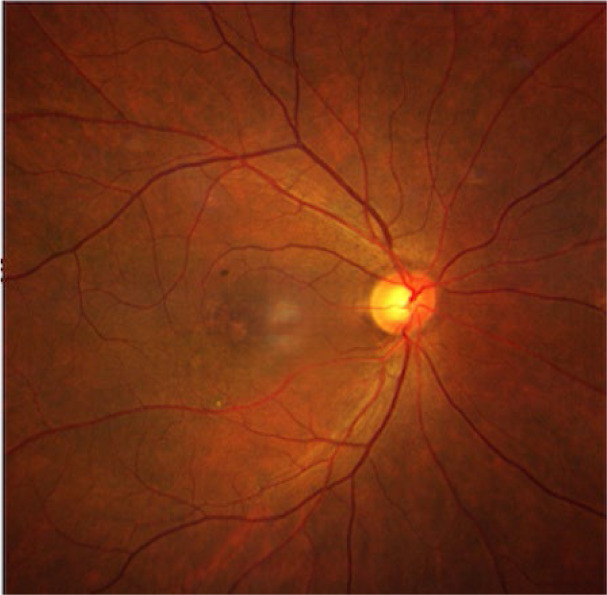
OD crowded disc (CDR 0.3) with well-defined margins and absence of edema or hemorrhages

Fundus (dilated): **Fig. 1** OD: Crowded disc with CDR 0.3; well-defined margins, no edema/hemorrhages; OCT radial: foveal thinning 193 μm, IS-OS disruptions and **2 A-D** OS: Hyperaemic 360°-disc edema with splinter hemorrhages; obliterated cup; peripapillary dot-blot hemorrhages; OCT RNFL 182 μm; 30-2 fields: 360° constriction (**Fig. 3A-B**).

**Fig. 2:**
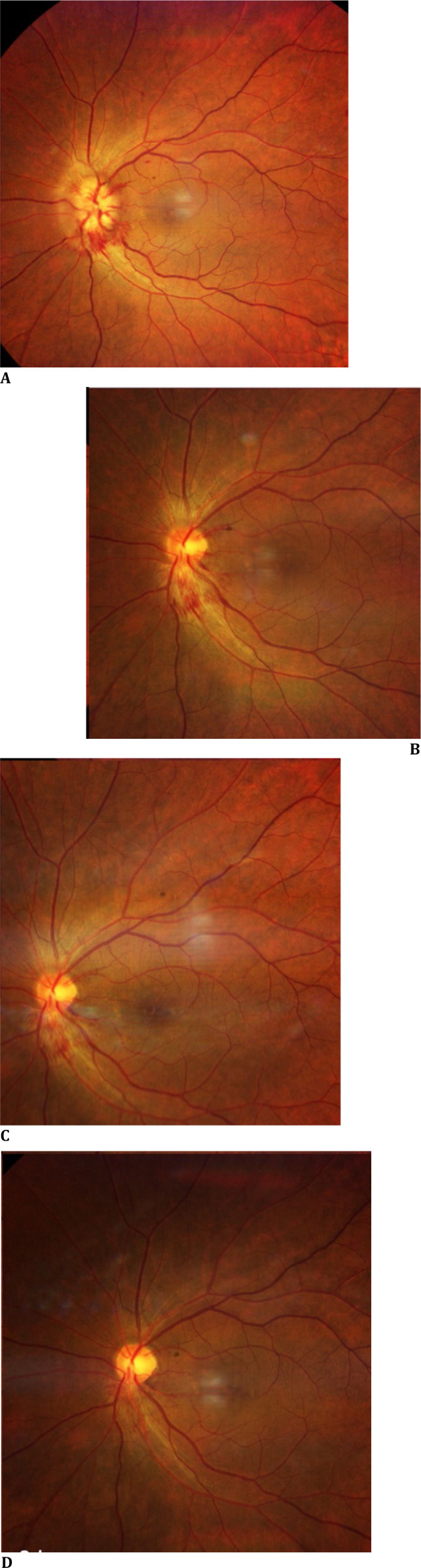
**A-D** Sequential OS color fundus images: (**A**) Day 1 showing hyperaemic 360°-disc edema with splinter hemorrhages; (**B**) Day 8 partial resolution; (**C**) Day 15 near-complete resolution; (**D**) Day 30 - disc pallor with CDR 0.2 (s/o crowded disc)

**Fig. 3:**
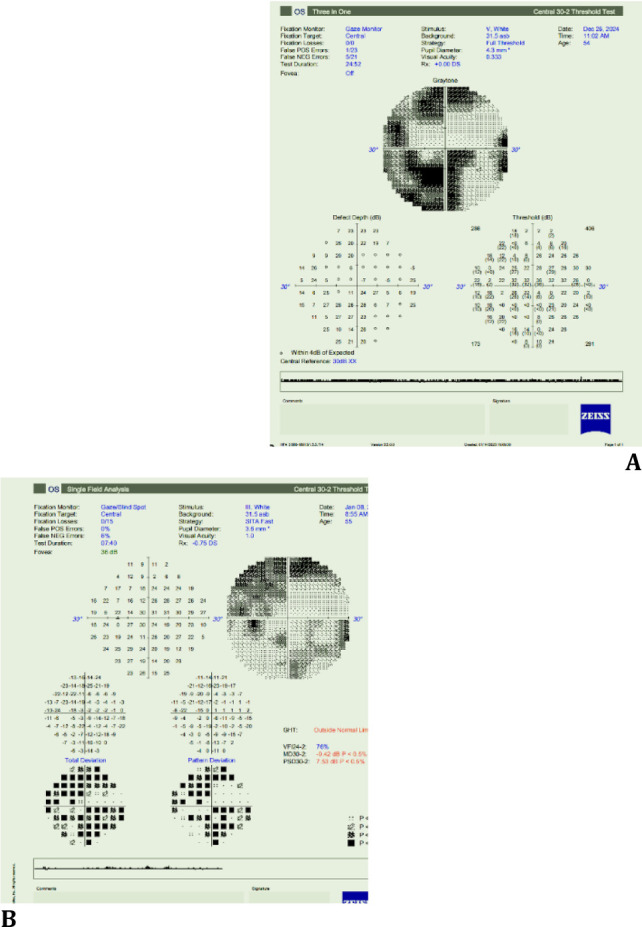
**A-B** 30-2 visual fields OS at presentation vs two-week follow-up, demonstrating marked improvement in field constriction

### Investigations

Blood: BP 126/82 mm Hg; PP2BS 146 mg/dL; lipids: elevated TG/LDL/VLDL; CBC normal; serum Ca normal; ESR elevated; VDRL nonreactive; smear normal.

Neuroimaging: HRCT brain & orbit: no SOL, demyelination, or compression.

B-Scan Ultrasonography: ONSD OS 6.02 mm.

**Fig. 4:**
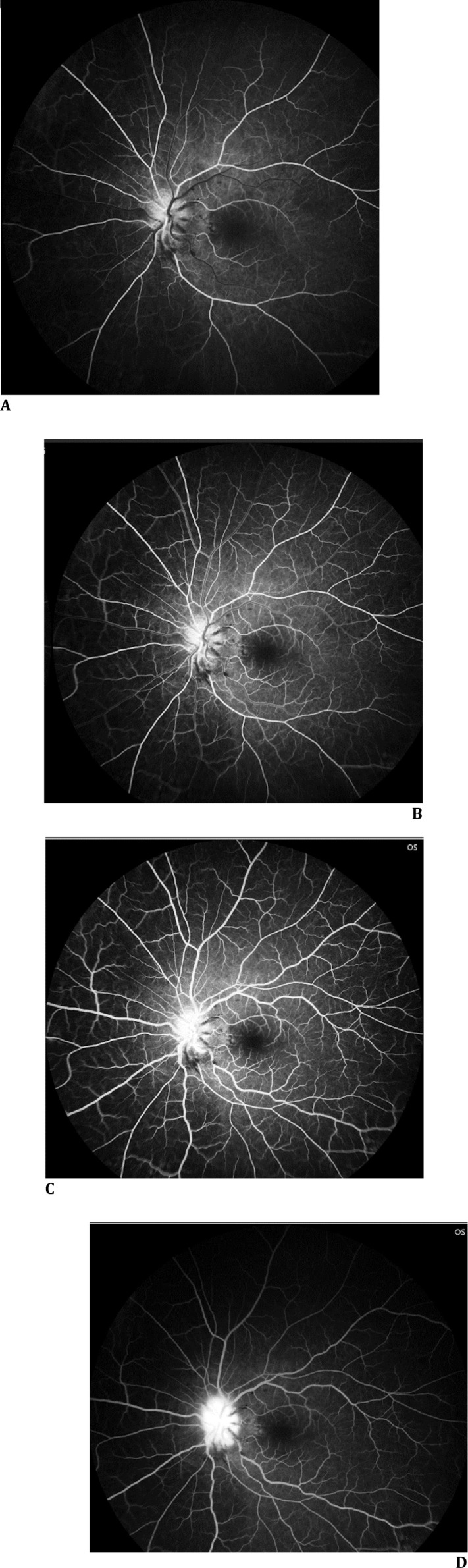
**A-D** FFA OS images: delayed arterial filling of disc, blocked fluorescence at hemorrhages, and late-phase leakage indicative of blood-retinal barrier breakdown

FFA: OD normal perfusion; OS delayed disc filling, blocked fluorescence at hemorrhages, late leakage (**Fig. 4A-D**).

### Differential Diagnosis

Primary: Incipient NAION

Differentials: Inflammatory optic neuropathy; compressive neuropathy (ruled out).

### Treatment

Aspirin 75 mg OD (vascular protection)

Prednisolone 1 mg/kg OD for one week (therapeutic inflammatory trial)

## Results

### Outcome and Follow-Up

One Week: OS edema resolved (RNFL 104 μm), OS vision 6/9, RAPD resolved; taper: 40 mg OD × 5 days, 30 mg OD × 5 days.

Two Weeks: Continued edema resolution, OS 6/6, marked field improvement.

Ten Weeks: On pred 5 mg OD + aspirin; no recurrence; advised systemic monitoring.

## Discussion

1. Anatomical Risk: Crowded Disc

A crowded fellow eye disc (CDR 0.3) constitutes a “disc at risk”, predisposing to NAION due to limited scleral canal space and axonal crowding [**1-4**]. This anatomical feature was evident in OD despite the absence of systemic vascular comorbidities.

2. Evidence for Systemic Steroids

Hayreh and Zimmerman’s open-label prospective RCT is the largest to evaluate steroids in NAION: treated patients achieved ≥2 lines of BCVA improvement in 69.8% vs. 40% in controls, with a pronounced benefit in those presenting with ≤20/70 [**2**]. Limitations included patient-driven enrollment and higher placebo uptake by uncontrolled diabetics, but it remains foundational for steroid use in NAION [**2**]. Our case’s rapid resolution of visual and edema findings mirrors these findings.

3. Structured Management Algorithm

To address variability in outcomes, we suggest:

Step 1: Evaluate risk factors (crowded disc, vascular profile).

Step 2: Conduct targeted investigations (ESR, VDRL, imaging).

Step 3: In equivocal cases with FFA leakage, initiate short-course steroids.

Step 4: Weekly monitoring of VA, OCT, RNFL, and visual fields; discontinue if no response.

Step 5: Long-term follow-up for vascular and inflammatory sequelae.

4. Risks and Limitations

Systemic steroids pose hyperglycemia, immunosuppression, and ocular side effects. Endocrine clearance and monitoring are mandatory. Not all NAION cases benefit; individualized, cautious application is crucial.

5. Clinical Implications

In low-resource settings, therapeutic steroid trials can guide diagnosis and management where advanced diagnostics are unavailable, provided rigorous selection and follow-up.

## Conclusion

This case demonstrates that rapid structural and functional recovery following steroids can help differentiate inflammatory optic neuropathy from incipient NAION when routine investigations are inconclusive. A crowded disc may predispose to NAION, yet should not preclude consideration of an inflammatory component. Short, carefully monitored steroid trials may serve as valuable diagnostic tools in resource-limited settings.

## References

[1] Hayreh SS (2013). Ischemic optic neuropathies – where are we now?. Graefes Arch Clin Exp Ophthalmol.

[2] Hayreh SS, Zimmerman MB (2008). Non-arteritic anterior ischemic optic neuropathy: role of systemic corticosteroid therapy. Graefes Arch Clin Exp Ophthalmol.

[3] Biousse V, Newman NJ (2009). Non-arteritic anterior ischemic optic neuropathy: clinical features, pathogenesis, and management. Lancet Neurol.

[4] Moorthy RS, Das T, Rao S (2010). The role of steroids in the management of inflammatory optic neuropathies. Indian J Ophthalmol.

[5] Figueroa MJ, Parisi F, Shields CL (2016). The role of fluorescein angiography in diagnosing optic neuropathy. JAMA Ophthalmol.

